# Clinical evaluation of the post-laminectomy syndrome in public hospitals in the city of São Luís, Brazil

**DOI:** 10.1186/s13104-015-1400-9

**Published:** 2015-09-17

**Authors:** João Batista Santos Garcia, Diego Parga Rodrigues, Diego Rafael Berbare Leite, Stephanie do Nascimento Câmara, Kenard da Silva Martins, Érica Brandão de Moraes

**Affiliations:** Anesthesiology, Pain and Palliative Care Department, Federal University of Maranhao, Av. São Marcos, n.4/502, Ponta da Areia, São Luís, MA 65077-310 Brazil

**Keywords:** Post-laminectomy pain syndrome, Herniated disc, Low back pain, Chronic pain, Neuropathic pain, Depression, Anxiety, Disability

## Abstract

**Background and objectives:**

Although not well known, post-laminectomy syndrome (PLS) is an important cause of chronic back pain, which may lead to decreased quality of life, disability and psychological disorders. This study aimed to evaluate the clinical characteristics and prevalence of PLS, to estimate its impact on the quality of life and to determine its association with anxiety, depression and disability in patients at public hospitals in São Luís, MA.

**Methods:**

Cross-sectional, descriptive and analytical study. Eighteen patients characterized as having PLS were selected, and their clinical, epidemiological and psychological characteristics, their quality of life and their levels of physical fitness were evaluated through clinical evaluations, Beck questionnaires, the Short Form-36 (SF-36), the Rolland-Morris questionnaire and the Douleur Neurophatique 4 questions. The multidimensional pain evaluation was performed using the McGill Pain Questionnaire.

**Results:**

The prevalence of post-laminectomy pain was 60 %. Most of the patients assessed in this study were male and received a family income of up to minimum wage; their mean age was 45 years. All of the patients presented with chronic, intense pain that had lasted an average of 7.22 years. The prevalence of neuropathic pain was 89.9 %. The physical appearance and functional capacity domains of the SF-36 were classified as unsatisfactory in 94.4 and 83.3 % of the patients, respectively. None of the patients exhibited high levels of physical fitness. The average score was 21.33 for anxiety and 18.88 for depression. There was a strongly positive and significant relationship between the anxiety and depression scores. Additionally, there was a moderately positive and significant relationship between the disability and anxiety scores. Regarding the correlation between the pain intensity and the quality of life, there was a moderately significant relationship between the patients’ mental health and their vitality.

**Conclusion:**

PLS exhibits a high prevalence and significance, and it causes high levels of morbidity in patients. Furthermore, PLS features intense levels of pain, reduced quality of life and greater physical and occupational disability.

**Electronic supplementary material:**

The online version of this article (doi:10.1186/s13104-015-1400-9) contains supplementary material, which is available to authorized users.

## Background

The pain associated with lumbar radiculopathy is caused by a combination of ischemia and inflammation of the nerve root, both of which are due to local pressure and to the release of inflammatory neurochemical factors present within the intervertebral disc [1].

The surgery to correct disc herniations is most commonly performed as an elective procedure in the patients who fail conservative treatment and who experience worsening of their lower limb pain and disability [2]. Studies indicate that the failure of herniated-disc surgeries is related to insufficient diagnoses and the incorrect selection of patients for these procedures [3, 4].

Lumbar laminectomy is one of the surgical procedures used to treat intervertebral disc (IVD) protrusions. It consists of removing the vertebral lamina to expose and allow access to the IVD that is responsible for the nerve root compression. Although the vast majority of lumbar sciatic pain is mechanical in nature, the surgical procedure is required in only 1–3 % of cases [5].

Post-laminectomy syndrome (PLS), or “Failed Back Surgery Syndrome” (FBSS), is defined by the *International Association for the Study of Pain* (IASP) as back pain, with or without referred or radiating pain, that is located mainly in the lower limbs, is of unknown origin and persists or begins after surgical procedures are performed to treat lumbar disc herniations [6]. Although not well known, PLS is an important cause of chronic back pain. Low back pain is a common complaint, with a reported peak prevalence in the adult population of 37 % and a lifetime prevalence between 60 and 85 %. In addition to the suffering and disability that PLS may inflict on patients, its impact on society is considerable [7].

Compared with other models of chronic pain, PLS patients with neuropathic pain experience intense levels of pain, lower quality of life, greater disability and higher rates of unemployment [7].

The pain stems from the involvement of various anatomical structures and manifests itself in various and unique ways in each patient [8]. Chronic pain is debilitating and often resistant to pharmacological, rehabilitational, psychotherapeutic and surgical treatments [9]. Chronic pain evokes emotions that may be equally disabling or even more disabling than the actual nosological condition that caused it. The pain can generate ideas about hypothetical losses and physical disability, which together can affect the emotional, social, cultural and family performance of the patient. Many patients’ lives begin to be solely centered on their pain. Their physical, mental and social deterioration—as well as the immobility caused by fear of exacerbating the pain—may result in a worsening of the associated mental illnesses and difficulties associated with their treatment. The difficulties that the patients encounter in understanding their problems and finding a cure, combined with a lack of coherent explanations from the professionals who assist them, destabilize and worsen their mental states.

Despite numerous studies addressing the prevention and treatment of PLS, little has been published nationally or internationally on the correlation between the anxiety, depression, disability and quality of life of individuals with PLS. We found no publications on this subject that are based in our region.

Therefore, the present study aimed to evaluate the clinical characteristics and the prevalence of PLS and to estimate its impact on the quality of life and its association with pain, anxiety, depression and disability in the patients at public hospitals in São Luís, Brazil.

## Methods

### Participants

We selected patients who had undergone at least one lumbar spine surgery to treat disc herniations in a period of 1 year and who did not show improvement of the original symptoms. The subjects were of legal age and were able to understand the questionnaires and to report their answers precisely.

The following participants were excluded from this study: patients with oncologic or chronic inflammatory diseases, pregnant women and individuals with severe psycho-behavioral impairments that could jeopardize the collection of the necessary information.

In the studied period, 188 spinal column surgeries were performed for various reasons at the reference hospitals of the public system of São Luís (President Dutra University Hospital, Dr. Carlos Macieira Hospital and Tarquinio Lopes Filho General Hospital). Of these surgeries, 73.9 % were performed at the University Hospital of the Federal University of Maranhão. The medical records of these patients were solicited, of which 18 (9.5 %) were not found. From the available medical records, 78 patients (45.9 %) were excluded from the study because they had undergone surgeries to treat etiologies (neoplasias, spinal cord injuries, congenital scoliosis) other than lumbar disc herniations. The remaining 92 patients had undergone the procedure of interest.

**Fig. 1:**
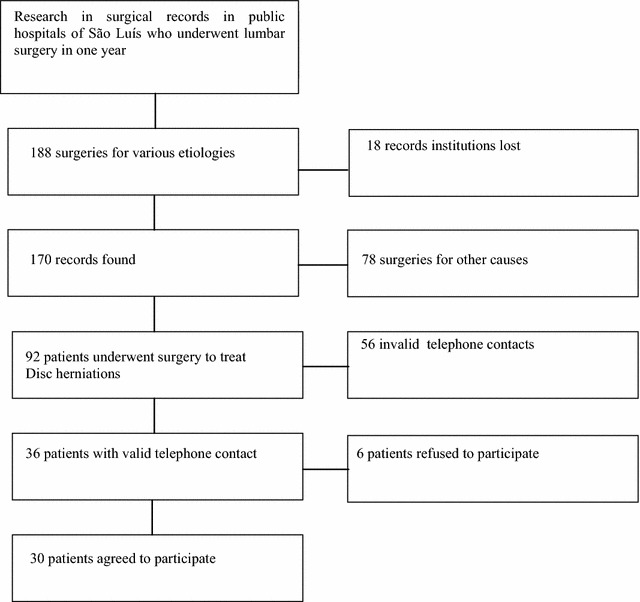
Flowchart of the study

Of these 92 patients, 32 lived in cities far from greater São Luís, and 24 had no working telephone number. Therefore, a total of 36 patients were analyzed. From these 36 patients, 6 (16.7 %) were lost from the study because they refused to participate in the survey or had changed their address. Consequently, 30 (83.3 %) patients were contacted, and only 18 met the inclusion criteria and remained in the sample for analysis (Fig. 1).

### Study design

The present investigation was a cross-sectional, descriptive and analytical study. The patients were contacted by telephone and were questioned regarding the persistence or worsening of their back pain after surgery. In cases where there was no improvement in the pain, the patient was invited to participate in the study. An appointment was scheduled at the outpatient clinic for chronic pain at the President Dutra University Hospital (“House of Pain”) under the supervision of doctors who were pain specialists.

The evaluation consisted of a clinical interview and the use of questionnaires validated for Brazil. The data were obtained by the researchers themselves, with support from the staff of the institution where the study was conducted and from the members of the Academic League of Pain of Maranhão.

### Instruments

The questionnaire Short Form-36 (SF-36) [10], which was validated for Brazil, was applied to estimate the quality of life as assessed according to eight domains (physical functioning, general health, physical appearance, emotional aspect, pain, mental health, social aspect and vitality) with scores ranging from 0 to 100. Higher scores reflected lower levels of impairment.

There are contradictions in the literature regarding the cutoff point of the SF-36 questionnaire; however, for the sake of this study, scores below 50 were considered as unsatisfactory, between 50 and 70 as satisfactory and equal to or greater than 70 as excellent.

Physical fitness was assessed using the Roland-Morris questionnaire [11], whose scale ranges from 0 to 24. The questionnaire is divided into three levels: at the 0–7 level, the individual exhibits a high level of physical fitness; at the 8–16 level, moderate fitness; and at the 17–24 level, poor fitness.

All of the patients were evaluated and followed up according to the assessment protocols for back pain that were written at the chronic pain department of the President Dutra University Hospital. The patients’ responses were documented regarding their pain’s onset, character, intensity, location, radiation, time of exacerbation, frequency, triggering factors, improvement factors, worsening factors and other associated characteristics; data were also collected from the neurological and musculoskeletal examinations.

The multidimensional characteristics of the pain were assessed by the reduced McGill Pain Questionnaire, which was validated for the Portuguese language [12]. The total number of descriptors and the pain index were analyzed based on the number and type of descriptors that were reported by the patients.

The presence of neuropathic pain was investigated using a DN4 questionnaire validated for the Portuguese language [13], for which patients with scores greater than or equal to 4 are regarded as having predominantly neuropathic pain.

The psychological evaluation aimed to quantify the patients’ anxiety and depression based on the Beck Inventory [14]. The original scale consists of 21 items, including symptoms and attitudes with intensities ranging from 0 to 3. The sum of the scores for each item reveals the intensity of the depression: values below 10 signify the absence of depression or the presence of mild depression, values between 10 and 18 signify mild to moderate depression, values between 19 and 29 signify moderate to severe depression, and values between 30 and 63 signify severe depression. For the measurement of anxiety, the sum of the items indicates the intensity as minimal (up to 10), mild (10–20), moderate (20–30) or severe (31–63).

### Statistical analysis

The analysis of the qualitative variables was performed using the absolute and relative frequencies and averages. Spearman’s correlation was used to correlate the various questionnaires and their components with the intensity of the pain. The following values were considered for the strength of association: strong (0.9–1), strong (0.75–0.9), moderate (0.5–0.75), weak (0.25–0.5) and very weak (0.0–0.25) [15]. Associations with a *p* value <0.05 were considered statistically significant. The data were analyzed using the STATA 10.0 software.

### Ethical aspects

All of the patients had previously signed an informed consent form and were examined by a multidisciplinary team. The appropriate follow-up was offered to these patients without incurring any additional burden to them. This work was approved by the University Hospital Research Ethics Committee in accordance with Resolution 196/96 under number 502/2009-80.

## Results

Of the 30 patients assessed, 18 reported persistent pain after surgery. Therefore, the prevalence of PLS was 60 % in the present study.

The mean age was 45 ± 12.46 (standard deviation, SD) years. Most of the patients had dark complexions (55.6 %), were male (66.7 %), were married (77.8 %), had up to a primary education (72.2 %) and earned a family income of up to minimum wage (50 %), as presented in Additional file 1: Table S1.

Only one patient reported that the pain did not affect his professional activity, and 27.8 % of the patients assessed were receiving health aid benefits. The majority reported not having changed jobs because of the pain (88.9 %).

All of the patients experienced chronic pain (greater than 3 months in duration), with an average duration of 7.22 years. The pain was predominantly of insidious onset (77.8 %), was moderately intense at the time of the appointment (38.9 %) (3.77 ± 3.15), was intense at its most severe point (83.3 %) (8.67 ± 2.00), was intense in general (50 %) (6.77 ± 2.48) and was continuous in its frequency (61.1 %) (Additional file 2: Table S2). The dermatomes most often related to the pain radiation were L5 (66.7 %) and S1 (72.2 %). The radiation of the pain was predominantly asymmetric (72.2 %). The most common periods of pain exacerbation were the early morning (33.3 %) and late afternoon (38.9 %), with a daily progressive evolution in 50 % of the patients. The most common types of pain were burning (55.6 %), twinging (66.7 %) and stabbing (72.2 %). Most of the patients reported difficulties with their movements (83.3 %) and sleep (61.1 %) due to the pain.

Muscular weakness was present in 61.1 % of the patients, and 88.9 % presented changes in the overall sensitivity of some of their affected segments during the physical examination. The majority exhibited altered reflexes (77.8 %), pain on flexion (88.9 %) and extension (72.2 %) of the lumbar spine and a positive Valsalva maneuver (16.7 %). The Lasègue maneuver was positive in only 22.2 %. The prevalence of neuropathic pain as assessed by the DN4 questionnaire was 89.9 % (6.1 ± 2.0 points) (Additional file 2: Table S2).

The multidimensional pain evaluation performed by the McGill Pain Questionnaire [12] revealed that the affective and evaluative domains were most affected, with mean potential descriptors of 76.6 % and 89 %, respectively (Additional file 3: Table S3).

When applying the SF-36 [10], the physical-appearance and functional-capacity domains were classified as unsatisfactory in 94.4 and 83.3 % of the patients, respectively. The mean values for each domain are shown in Additional file 4: Table S4.

From the Beck inventories, the mean score for anxiety was 21.33 ± 14.49, out of a total of 63 possible points (Additional file 2: Table S2). Of these patients, 55.6 % were classified as having moderate or severe anxiety. The average score for depression was 18.88 ± 11.98, out of a total of 63 possible points (Additional file 2: Table S2). Of these patients, 50 % were classified as having moderate to severe depression.

The mean positive response obtained using the Roland-Morris questionnaire [11] was 17.39 ± 4.31, revealing major physical disabilities in this population (Additional file 2: Table S2). Most of the patients (61.1 %) were classified as having poor physical fitness. No individual was classified as having a high level of physical fitness.

After comparing the pain intensity, the Beck scores for anxiety and depression and the Rolland-Morris Disability Questionnaire (Additional file 5: Table S5), a strongly positive and significant relationship was identified between the anxiety and depression scores. Additionally, there was a moderately positive and significant relationship between the Rolland-Morris [11] questionnaire scores and the anxiety scores.

After comparing the pain intensity and the SF-36 items (Additional file 6: Table S6), a moderately positive and significant relationship was observed between the mental health and vitality items of the SF-36 questionnaire.

## Discussion and conclusions

The prevalence of persistent pain after the surgical treatment of lumbar disc herniations was high in our study population, demonstrating a prevalence rate higher than that reported in the literature [7]. As many as one-third of the patients undergoing surgery for the correction of lumbar disc conditions experience recurrent postoperative symptoms [16]. Walker [17] states that 20–40 % of the patients undergoing lumbar surgery will not experience benefits from the procedure and that the condition will worsen in 1–10 %. The high prevalence in our study was perhaps due to inaccurate indications for surgery, in which the preoperative pain may have been attributed to disc herniations despite the other possible differential diagnoses. The main causes of PLS are foraminal stenosis, internal disc extrusion, pseudoarthrosis and neuropathic pain, which in combination account for more than 70 % [18] of the cases.

Several authors [18, 19] have suggested that the misinterpretation that a herniated disc is causing low back pain is the most common reason behind the spinal surgeries that result in post-laminectomy chronic pain syndrome beginning immediately after the procedures. This misinterpretation may be partially caused by an overestimation of the anatomical findings that are revealed during the imaging evaluations but are not related to the lumbago and usually do not explain the pain or justify surgical intervention. The overvaluation of complementary exams by contemporary medicine might be responsible for the high prevalence of PLS. Hasty diagnoses using imaging methods rather than clinical observations can lead to unnecessary treatments (including surgery) that, in turn, cause iatrogenic conditions. Iatrogenic PLS might soon become common and thus would require the incorporation of a new mentality in current medical practice that includes the concept of quaternary prevention.

The background of the population in the present study indicated a low socioeconomic status and predominantly consisted of men who were still in their productive years, despite the fact that many were out of work and were receiving health-aid benefits. Rodriguez Garcia [20] found an equal prevalence of the PLS in both genders, unlike other studies that have determined a slightly higher prevalence among males. A study conducted in Brazil has determined that most PLS patients are middle-aged males and that the mean duration of their symptoms is 96 months [19]. These results corroborate those of the present study and indicate the significance of the syndrome in males and in the middle-aged population, which are common categories of patients with lumbar disc herniations.

The analysis of our case series demonstrated that 38.9 % of the patients were unemployed, which is slightly higher than the rate that is reported in the literature [20]. This result is most likely caused by the socio-cultural differences between our region and those examined in other studies, such as differences such as the culture of labor accommodation and the dependency of the local population on the welfare system.

The various clinical manifestations of PLS often overlap and have lumbago as a common characteristic [20]. The following signs may be revealed by neurological examinations: hypoesthesia; hyperalgesia; hyperpathia or mechanical allodynia; motor impairment; impairment of the myotatic reflexes; and trophic abnormalities in the lumbar, gluteal, MMII and/or neurovegetative regions, such as tissue-perfusion and temperature-regulation impairments that may include fecal/urinary incontinence and impaired sexual performance. In PLS, the pain may be musculoskeletal, neuropathic or mixed [21].

The clinical characteristics of the pain in our patients were consistent with the literature. All of the patients experienced chronic pain, which was predominantly of moderate intensity at the time of examination. The vast majority of the patients presented a neuropathic pain profile based on the analysis of the DN4 questionnaire. This result, coupled with the chronicity component, would influence the diagnosis and management of these patients and the refractoriness of their pain, causing it to be characterized as a disease. Reduced muscle strength, sensitivity and deep reflexes were present in most patients during their physical examinations. These characteristics contributed to the disability exhibited by these individuals and might have represented somatization disorders in search of secondary gains. These assumptions help to confirm the complexity of the subject and the need for more studies to clarify the topic.

A detailed psychiatric evaluation revealed that most of the PLS patients had defined psychiatric diagnoses, were suffering from personality disorders, had normal pre-pain personalities or suffered from reactive depression [21]. In agreement with this finding, the analysis of the patients in the present study revealed a high prevalence of anxiety and depression, with a high average score based on the Beck inventory. These results confirm the influence of chronic pain on the process of mental health, which requires psychological and/or psychiatric counseling for the management of these individuals to obtain satisfactory results in their treatment.

The anxiety and depression scores showed a strong correlation in the present study, suggesting that there is an intimate relationship between a combination of psychiatric disorders, especially mood disorders, in patients with chronic pain. Anxiety and depression are manifestations of the same spectrum of disease and characterize the intense suffering of individuals who are living with persistent pain, particularly those patients who have had previous surgeries as a failed attempt to solve their symptoms—as has occurred with PLS.

Mental health impairment maybe influences a patient’s sense of well-being, as demonstrated in our results by the positive relationship between mental health and vitality, which was measured by the SF-36 questionnaire. This relationship sensitizes us to a greater appreciation of the emotional component of the syndrome and can be a key component for the effective control of PLS. Preoperative and postoperative psychological evaluations as well as a follow-up for these patients may be appropriate measures for future attempts to control the pathophysiologic processes involved in the syndrome.

According to some authors [21], the quality of life is clearly affected in all aspects of the SF-36 in the assessed individuals who have presented with postoperative pain after the repair of lumbar hernias. Our study confirms this finding, revealing greater impairments in these patients’ functional capacity, physical aspect and pain. Consequently, there is a reduced quality of life, with the pain becoming intricately involved in the lives of these individuals and negatively influencing almost all of their daily activities and functions. Most of the patients in our study had difficulties sleeping and performing movements because of their pain. This finding was reflected by a higher frequency of the affective and evaluative components in the multidimensional assessment of pain by the McGill Pain Questionnaire.

A study conducted in Maine, USA, concluded that optimal results of spinal column surgery (regarding patient pain and function) occurred in areas with the lowest rates of surgery, whereas the worst outcomes occurred in areas with the highest rates of surgery [7]. Improvements in analgesia, function, quality of life and satisfaction were significantly higher in regions with lower rates of surgery. These data corroborate the findings in the present study, as revealed by the profound physical disabilities in our population. No patient in the present study was classified as having a high level of physical fitness based on the Roland-Morris questionnaire. From these results, it can be hypothesized that surgery does not always yield satisfactory results for individuals because the procedures do not return the individuals to their normal functions in society. This physical disability often leads to greater consequences (as demonstrated here by the positive correlation with anxiety), transforming a single problem into a multidimensional process with major social implications.

It should also be noted that this subject is controversial. Some investigations have shown beneficial results from surgery. A prospective non-randomized observational study [22] suggests that patients who suffer from low back pain caused by lumbar disc herniations and who undergo surgery exhibit positive responses to treatment, as do those patients who are treated conservatively. The same investigation has also demonstrated that patients who opt for interventional treatment report significantly greater improvements than those who elect nonsurgical treatments; however, the study was non-randomized and supported only by subjective reports from the patients, warranting a careful interpretation of the results. However, a controlled and randomized cohort study [23] has demonstrated that patients with disc herniations who are treated surgically experience significantly greater improvements in their pain, function, satisfaction and self-assessments of their progress over 4 years of follow-up compared with patients who are treated conservatively, despite worse results for the motor activity in the former group after surgery.

The main limitation of the present study was the small number of patients who were assessed. This restriction was due to the loss of patient contact by telephone and to the failure of some patients to meet the inclusion criteria. Another obvious limiting factor was the possible lack of follow-up and rehabilitation of these patients after surgery, which was caused by the inadequate management of the responsible medical team, by a public health system that was deficient in the rehabilitation sector or by obstacles (such as difficulty in movement) that were inherent to the patients. These factors negatively contributed to the postoperative pain control in the long term and were reflected in the results observed in this study. Future longitudinal studies are needed to better understand the relation among all these evaluated factors and pain.

Despite evident progress in the treatment of degenerative diseases of the spine, many problems remain unresolved. Furthermore, the rate of PLS after surgical decompression of the nerve root remains considerably high [22, 23], causing severe pain, impaired quality of life, physical or motor disabilities and psychological disorders. We propose that investments be made in the prevention and interdisciplinary management of afflicted patients to reduce the incidence and morbidity related to this disease and to lessen the commensurate negative impacts on these individuals and their societies.

## Additional files

**Additional file 1: Table S1.** Sociodemographic characteristics of patients undergoing lumbar laminectomy in public hospitals of São Luis, Brazil.
**Additional file 2: Table S2.** Scores of Questionnaires DN4, Rolland Morris, Beck (anxiety and depression) and Numerical Pain Scale applied in patients undergoing lumbar laminectomy in public hospitals of São Luís, Brazil.
**Additional file 3: Table S3.** Score of McGill Questionnaire scores distributed in patients undergoing lumbar laminectomy in public hospitals of São Luís, Brazil.
**Additional file 4: Table S4.** Average score for each domain of SF-36 Questionnaire applied in patients undergoing lumbar laminectomy in public hospitals of São Luís, Brazil.
**Additional file 5: Table S5.** Correlation between pain intensity, anxiety and depression scores and disability (Rolland Morris) in patients undergoing lumbar laminectomy in public hospitals of São Luís, Brazil.
**Additional file 6: Table S6.** Correlation between pain intensity and items of SF36 in patients undergoing lumbar laminectomy in public hospitals of São Luís, Brazil.
