# Supplementary material for: Clinical evaluation of the post-laminectomy syndrome in public hospitals in the city of São Luís, Brazil
Source: BMC Res Notes. 2015 Sep 17;8:451. doi: 10.1186/s13104-015-1400-9 (PMC4574019; doi:10.1186/s13104-015-1400-9)
Supplement: Supplementary file 1 — Additional file 1: Table S1. Sociodemographic characteristics of patients undergoing lumbar laminectomy in public hospitals of São Luis, Brazil. [file 13104_2015_1400_MOESM1_ESM.docx]

Table S1 - Sociodemographic characteristics of patients undergoing lumbar laminectomy in public hospitals of São Luis, Brazil.

| Variable | n(%) |
| --- | --- |
| Age | 45(12,46)* |
| Marital status |  |
| Married | 14 (77,7) |
| Single | 4 (22,2) |
| Divorced | 0 (0,0) |
| Widower | 0 (0,0) |
| Color |  |
| White | 5 (22,2) |
| Black | 3 (16,6) |
| Brown | 10 (61,1) |
| Gender |  |
| Male | 12 (66,6) |
| Female | 6 (33,3) |
| Educational level (years) |  |
| ≤ 4 | 2 (11,1) |
| 4 to 8 | 11 (61,1) |
| ≥ 8 | 5 (27,7) |
| Occupational status |  |
| Active | 11 (62,1) |
| Inactive | 7 (38,8) |
| Income |  |
| < 250 US$ | 9 (50,0) |
| 250 to 500 US$ | 9 (50,0) |
| > 500 US$ | 0 (0,0) |

*Average and Standard Deviation
