# Supplementary material for: Clinical evaluation of the post-laminectomy syndrome in public hospitals in the city of São Luís, Brazil
Source: BMC Res Notes. 2015 Sep 17;8:451. doi: 10.1186/s13104-015-1400-9 (PMC4574019; doi:10.1186/s13104-015-1400-9)
Supplement: Supplementary file 2 — Additional file 2: Table S2. Scores of Questionnaires DN4, Rolland Morris, Beck (anxiety and depression) and Numerical Pain Scale applied in patients undergoing lumbar laminectomy in public hospitals of São Luís, Brazil. [file 13104_2015_1400_MOESM2_ESM.docx]

Table S2. Scores of Questionnaires DN4, Rolland Morris, Beck (anxiety and depression) and Numerical Pain Scale applied in patients undergoing lumbar laminectomy in public hospitals of São Luís, Brazil.

| Variable | n | Average ± SD | | Minimum Score | Maximum Score |
| --- | --- | --- | --- | --- | --- |
| DN4 | 18 | 6,11 | ±2 | 2 | 9 |
| Rolland Morris(Total) | 18 | 17,38 | ±4,31 | 11 | 24 |
| Beck Anxiety | 18 | 21,33 | ±14,49 | 0 | 63 |
| Beck Depression | 18 | 18,89 | ±11,97 | 4 | 48 |
| Pain intensity (NS) | 18 | 6,77 | ±2,48 | 1 | 10 |

SD:Standard deviation
