# Supplementary material for: Clinical evaluation of the post-laminectomy syndrome in public hospitals in the city of São Luís, Brazil
Source: BMC Res Notes. 2015 Sep 17;8:451. doi: 10.1186/s13104-015-1400-9 (PMC4574019; doi:10.1186/s13104-015-1400-9)
Supplement: Supplementary file 3 — Additional file 3: Table S3. Score of McGill Questionnaire scores distributed in patients undergoing lumbar laminectomy in public hospitals of São Luís, Brazil. [file 13104_2015_1400_MOESM3_ESM.docx]

Table S3. Score of McGill Questionnaire scores distributed in patients undergoing lumbar laminectomy in public hospitals of São Luís, Brazil.

| Domain | Average + SD | % Relative to the maximum possible items | Index Pain |
| --- | --- | --- | --- |
| Sensory | 7,33 ± 2,95 | 73,3% | 15,16 ± 8,30 |
| Affective | 3,83 ± 1,62 | 76,6% | 6,06 ± 3,47 |
| Evaluative | 0,89 ± 0,32 | 89% | 2,50 ± 1,65 |
| Miscellany | 2,78 ± 1,31 | 69,5% | 5,72 ± 3,44 |
| Total | 14,83 ± 5,63 | 74,2% | 29,44 ± 15,23 |

SD:Standard deviation
