# Supplementary material for: Clinical evaluation of the post-laminectomy syndrome in public hospitals in the city of São Luís, Brazil
Source: BMC Res Notes. 2015 Sep 17;8:451. doi: 10.1186/s13104-015-1400-9 (PMC4574019; doi:10.1186/s13104-015-1400-9)
Supplement: Supplementary file 4 — Additional file 4: Table S4. Average score for each domain of SF-36 Questionnaire applied in patients undergoing lumbar laminectomy in public hospitals of São Luís, Brazil. [file 13104_2015_1400_MOESM4_ESM.docx]

Table S4. Average score for each domain of SF-36 Questionnaire applied in patients undergoing lumbar laminectomy in public hospitals of São Luís, Brazil

| Domain | N | Average | SD (±) | Minimum score | Maximum score |
| --- | --- | --- | --- | --- | --- |
| Functional Capacity | 18 | 26,95 | ±16,72 | 0 | 55 |
| Physical appearance | 18 | 9,72 | ±19,43 | 0 | 75 |
| Pain | 18 | 28,95 | ±16,08 | 0 | 52 |
| General health | 18 | 43,05 | ±21,08 | 5 | 70 |
| Vitality | 18 | 44,45 | ±16,88 | 15 | 70 |
| Social Aspect | 18 | 54,16 | ±30,01 | 12,5 | 100 |
| Emotional Aspect | 18 | 25,77 | ±35,23 | 0 | 100 |
| Mental Health | 18 | 41,77 | ±22,17 | 0 | 72 |

SD:Standard deviation
