# Supplementary material for: Clinical evaluation of the post-laminectomy syndrome in public hospitals in the city of São Luís, Brazil
Source: BMC Res Notes. 2015 Sep 17;8:451. doi: 10.1186/s13104-015-1400-9 (PMC4574019; doi:10.1186/s13104-015-1400-9)
Supplement: Supplementary file 5 — Additional file 5: Table S5. Correlation between pain intensity, anxiety and depression scores and disability (Rolland Morris) in patients undergoing lumbar laminectomy in public hospitals of São Luís, Brazil. [file 13104_2015_1400_MOESM5_ESM.docx]

Table S5. Correlation between pain intensity, anxiety and depression scores and disability (Rolland Morris) in patients undergoing lumbar laminectomy in public hospitals of São Luís, Brazil.

|  | Pain Intensity | Beck depression | Beck Anxiety | Rolland Morris |
| --- | --- | --- | --- | --- |
| Pain Intensity | 1 |  |  |  |
| Beck Depression | 0,2858 | 1 |  |  |
| Beck Anxiety | 0,2316 | 0,8104* | 1 |  |
| Rolland Morris | 0,1160 | O,7164 | 0,7287* | 1 |

*p<0,05
