# Supplementary material for: Clinical evaluation of the post-laminectomy syndrome in public hospitals in the city of São Luís, Brazil
Source: BMC Res Notes. 2015 Sep 17;8:451. doi: 10.1186/s13104-015-1400-9 (PMC4574019; doi:10.1186/s13104-015-1400-9)
Supplement: Supplementary file 6 — Additional file 6: Table S6. Correlation between pain intensity and items of SF36 in patients undergoing lumbar laminectomy in public hospitals of São Luís, Brazil. [file 13104_2015_1400_MOESM6_ESM.docx]

Table S6. Correlation between pain intensity and items of SF36 in patients undergoing lumbar laminectomy in public hospitals of São Luís, Brazil.

|  | Pain intensity | Functional Capacity | Physical appearance | Pain | General health | Vitality | Social Aspect | Emotional aspect | Mental health |
| --- | --- | --- | --- | --- | --- | --- | --- | --- | --- |
| Pain intensity | 1 |  |  |  |  |  |  |  |  |
| Functional Capacity | -0,3343 | 1 |  |  |  |  |  |  |  |
| Physical appearance | -0,4399 | 0,2916 | 1 |  |  |  |  |  |  |
| Pain | -0,0097 | 0,4034 | 0,3112 | 1 |  |  |  |  |  |
| General health | 0,0567 | -0,1166 | -0,2035 | 0,0350 | 1 |  |  |  |  |
| Vitality | 0,1782 | -02229 | -0,133 | 0,0121 | 0,1311 | 1 |  |  |  |
| Social Aspect | -0,4650 | 0,2881 | 0.1382 | 0,1825 | 0,1383 | 0,1628 | 1 |  |  |
| Emotional aspect | -0,1450 | 0,3893 | 0,1937 | 0,1933 | 0,1365 | -0,1552 | 0,1713 | 1 |  |
| Mental health | 0,1930 | 0,1063 | -0,2067 | 0,0769 | 0,03446 | 0,7453* | 0,2016 | 0,2435 | 1 |

*p<0,05
